# YAP and the RhoC regulator ARHGAP18, are required to mediate flow-dependent endothelial cell alignment

**DOI:** 10.1186/s12964-020-0511-7

**Published:** 2020-02-03

**Authors:** Paul R. Coleman, Angelina J. Lay, Ka Ka Ting, Yang Zhao, Jia Li, Sorour Jarrah, Mathew A. Vadas, Jennifer R. Gamble

**Affiliations:** Centre for the Endothelium, Vascular Biology Program, Centenary Institute, The University of Sydney, Locked Bag 6, Newtown, Sydney, 2042 Australia

**Keywords:** ARHGAP18, Endothelial cells, YAP, Mechanotransduction, Inflammation

## Abstract

**Background:**

Vascular endothelial cell alignment in the direction of flow is an adaptive response that protects against aortic diseases such as atherosclerosis. The RhoGTPases are known to regulate this alignment. We have shown previously that ARHGAP18 in endothelial cells is a negative regulator of RhoC and its expression is essential in flow-mediated alignment. Depletion of ARHGAP18 inhibits alignment and results in the induction of a pro-inflammatory phenotype. In embryogenesis, ARHGAP18 was identified as a downstream effector of the Yes-associated protein, YAP, which regulates cell shape and size.

**Methods:**

We have used siRNA technology to deplete either ARHGAP18 or YAP in human endothelial cells. The in vitro studies were performed under athero-protective, laminar flow conditions. The analysis of YAP activity was also investigated, using high performance confocal imaging, in our ARHGAP18 knockout mutant mice.

**Results:**

We show here that loss of ARHGAP18, although decreasing the expression of YAP results in its nuclear localisation consistent with activation. We further show that depletion of YAP itself results in its activation as defined by an in increase in its nuclear localisation and an increase in the YAP target gene, CyR61. Depletion of YAP, similar to that observed for ARHGAP18 depletion, results in loss of endothelial cell alignment under high shear stress mediated flow and also in the activation of NFkB, as determined by p65 nuclear localisation. In contrast, ARHGAP18 overexpression results in upregulation of YAP, its phosphorylation, and a decrease in the YAP target gene Cyr61, consistent with YAP inactivation. Finally, in ARHGAP18 deleted mice, in regions where there is a loss of endothelial cell alignment, a situation associated with a priming of the cells to a pro-inflammatory phenotype, YAP shows nuclear localisation.

**Conclusion:**

Our results show that YAP is downstream of ARHGAP18 in mature endothelial cells and that this pathway is involved in the athero-protective alignment of endothelial cells under laminar shear stress. ARHGAP18 depletion leads to a disruption of the junctions as seen by loss of VE-Cadherin localisation to these regions and a concomitant localisation of YAP to the nucleus.

## Background

The alignment of endothelial cells [1] in the direction of blood flow is a response to the haemodynamic shear stress and is mediated through mechanosensors including integrins, the PECAM/VE-cadherin/VEGFR complex and caveolae. Such signals then act on the small RhoGTPases, important molecular switches for the regulation of many cellular processes, including cell shape, to mediate the flow-induced alignment process [2]. In particular RhoA and RhoC have been shown to be critical in this shape change of ECs. Importantly such alignment suppresses the inflammatory nature of the endothelium and is said to be athero-protective [3].

The activity of the GTPases are regulated by three mediators, guanine nucleotide exchange factors [4] which activate GTPases, GTPase-activating proteins [4] that de-activate the activity, and guanine nucleotide-dissociation inhibitors [4] that sequestrate the GTPase [4]. P190RhoGAP acting downstream of the integrin beta1/caveolin axis controls RhoA, in the formation of stress fibres and adaptation of ECs in response to flow [5]. The GEF, RacGEFTrio is essential for maintaining active Rac at the downstream area of the aligning cell but interestingly this is in a GEF independent manner [6]. This is consistent with the growing data showing that the GAPs and GEFs can exhibit both GAP/GEF dependent and independent functions. Most recently, we have shown ARHGAP18, a regulator of RhoC in ECs [7] is critical in flow mediated cell alignment, and as such its expression is essential to limit the development of atherosclerosis [8]. In a GAP independent manner, ARHGAP18 (a.k.a *SENEX*) promotes ECs senescence and the senescence is unique in showing a predominantly anti-inflammatory phenotype [9, 10] mediated through caveolae up-regulation [1]. Furthermore, ARHGAP18 expression is essential for ECs survival under stress, it localises to microtubules, is involved in the stabilisation of ECs cell junctions and for limiting angiogenic sprouting [7, 11]. In smooth muscle cells ARHGAP18 again displays an anti-inflammatory phenotype, as it inhibits the contractile to synthetic conversion, maintains the associated anti-inflammatory phenotype and limits thoracic aortic aneurysm formation [12]. Therefore, we suggest that ARHGAP18 limits or curbs the inflammatory nature of the vasculature, acting as a vascular protective gene.

The Yes-associated protein (YAP), a transcriptional coactivator of the HIPPO family, is a mechanotransduction protein that controls proliferation, cell shape and organ size [13–15]. Recently YAP has also been linked to the response of ECs to laminar flow [16] and YAP activation can promote expression of an inflammatory gene profile [17]. The regulation of the activity of YAP is complex. YAP is continually shuttling between the nucleus and the cytoplasm with nuclear localisation a key determinant of its activation to regulate downstream pro-inflammatory target genes. Phosphorylation at key serine residues (eg S127) is required for cytoplasmic translocation and 14–3-3 binding with eventual ubiqitination and degradation [18, 19]. However, S127-phosphorylated YAP is also found in the nucleus suggesting further regulatory events to control its localisation [20]. RhoGTPases lie both upstream and downstream of YAP. In embryogenesis, ARHGAP18 is an effector of YAP to regulate cell shape [21]. Therefore, we investigated whether the YAP/ARHGAP18 axis is part of the mechanotransduction cascade in ECs to respond to shear stress.

## Materials and methods

### In vitro shear flow experiment

Human umbilical vein endothelial cells (HUVEC) were isolated and maintained in 5% CO_2_ incubator according to the established method [22]. HUVEC were used at passage 1–3 for all experiments. Fifty thousand cells were cultured on IBIDI slides for 24 h to allow attachment then either kept as static controls or expose to various shear stress conditions using IBIDI pump system (IBIDI GmbH, Germany). Protein expression studies were performed using protein samples from HUVEC cultured under laminar (20dyne/cm^2^) or oscillating/disturbed (2dyne/cm^2^/1 Hz) flow.

### RhoGTPase activity assay

Measurement of active RhoA and RhoC levels was determined using the GLISA assays (Cytoskeleton) as previously described [7]. Briefly, control or YAP siRNA treated HUVECs were incubated for 1 h in serum free medium 199, followed by incubation in medium with 1% serum overnight. Cells were stimulated with TNF-α for 1 h and lysed for the measurement of RhoA and RhoC activity according to the manufacturer’s protocol.

### Immunofluorescence staining

For immunofluorescence staining of IBIDI slides or EZ cell (Merck), slides were fixed for 15 min with 4% paraformaldehyde, permeabilised for 10 min with 0.1%Triton X-100 in PBS followed by 2 h blocking with 2% BSA before probing with various antibodies. Primary antibodies and concentrations used were: Mouse Monoclonal anti-ARHGAP18 (clone 2A3-F, in-house, 5 μg/mL), Rhodamine Phalloidin (R415, Life Technologies), Rabbit Monoclonal anti-NF-κB p65 (C22B4, Cell Signaling), Rat Polyclonal anti-VE-cadherin (AF938, R&D Systems) and Rabbit Monoclonal anti- YAP (14,074, Cell Signaling).

### *En face* immunofluorescence stain of aortic tree

Eight to 10 weeks old male mice were euthanized and pressure perfused with saline through the left ventricle followed by perfusion with 5 ml of chilled 4% paraformaldehyde [23] solution. The aortic tree was carefully dissected and opened longitudinally to expose the lumen. The tree was then sandwiched between two glass slides and fixed in 4% PFA for another 2 h on ice followed by an overnight blocking with PBS containing 1% BSA. All primary antibodies were prepared in 4 ml of PBS containing 1% BSA and 5% normal serum. The following primary antibodies were used, Rat anti Mouse CD144 (555,289, BD Biosciences), and Rabbit Monoclonal anti-YAP (14,074, Cell Signaling). Samples were washed with PBS/Tween20 for 2 h (12x10min washes). All secondary antibody were used at 1:2000 dilution and incubated for 2 h at room temperature and washed for 2 h before staining with DAPI for 10 min. Aorta with lumen facing up were mounted and cover slipped using Prolong Gold. Images were captured at 63X using Confocal microscope (Leica TCS SP5) using a HCX PL APO Lbd Bl 63x/1.40–0.60 NA objective. All images for p65, eNOS, ICAM and ARHGAP18 were captured at the endothelial cell layer as identified by VE-Cadherin staining.

### Image analysis YAP nuclear localization

Total YAP expression and nuclear localization in the aortic laminar region were examined using confocal microscopy (Leica SP5 model x) and magnification of 630x. A total of 6 fields of view were taken for each mouse per group and analysed using the ImageJ software (version 1.49 m). For the analysis, each z-stacked image was split into 3 channels of VE-Cadherin, YAP and DAPI. As total YAP was reduced in the KO mice, the threshold values for YAP in the WT and ARHGAP18 KO were set to a range of 34 to 40 with dark background. The image calculator with the function “multiply” between DAPI and YAP channels from each field of view was used to isolate nuclear areas that were YAP positive. Total number of endothelial cells per view was calculated using VE-Cadherin expression as reference. The percentage of nuclear positive YAP cells per field was calculated using number of nuclear YAP/total EC number.

### siRNA methodology

For ARHGAP18 knockdown, HUVECs were transfected with either stealth siRNA control (low GC content), 5 nM) or two stealth siRNAs (HSS132562, HSS190252; 5 nM; Life Technologies) using Lipofectamine RNAiMAX (Life Technologies) as previously described [7]. Reagents for YAP knockdown were from Thermo Fisher (SI02662954, SI00084567, SI04438637 and SI04438644) and TAZ knockdown were purchased from QIAGEN (1,027,416, 5 nM).

### Western blot analysis

Total cell lysates were prepared from HUVECs exposed to various experimental conditions. Equal amount of total proteins (8 μg) were loaded and separated on 4–12% NuPAGE gradient gel (Life Technologies), transferred to PVDF membrane and detect with Enhanced Chemiluminescence substrate (Pierce) as per the manufacturer’s instructions. Primary antibodies for YAP (14,074, Cell Signaling), YAP-PhosphoSer127 (ab76252, Abcam), VCAM1 (ab134047, Abcam), eNOS (610,297, BD Biosciences), Rabbit anti-CYR61 (144,795, Cell Signaling) and ARHGAP18 clone 2A3-F3 (in-house), were used at 1:500–1000 and Actin-HRP (Abcam) was used at 1:10,000 dilution in PBS/Tween20 containing 1% BSA.

### Statistical analysis

All calculated values represent means ± SD. Statistical significances was determined by two way ANOVA with poshoc analysis or Unpaired two tailed Student *t*-test was used in some instances to compare means. Mean differences with *p* value of < 0.05 was considered statistically significance.

## Results

### In mature EC ARHGAP18 regulates YAP expression

**Fig. 1:**
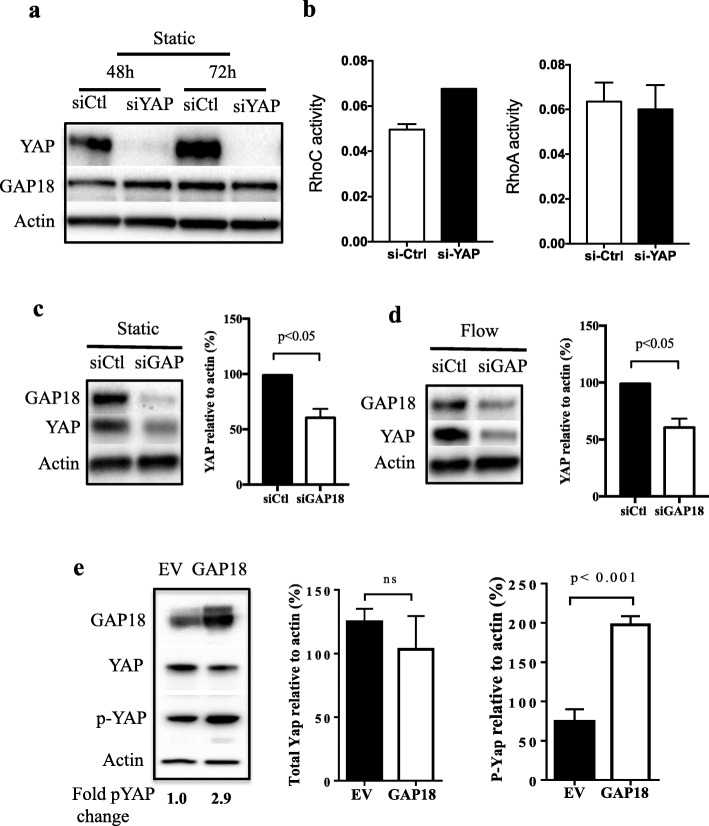
YAP is downstream of ARHGAP18. **a** Deletion of YAP (siYAP) did not affect ARHGAP18 expression under static conditions measured 48 h and 72 h post transfection. Representative Western blot of 3 experiments. **b** YAP depletion increased activity of RhoC but not RhoA when measured after 48 h of depletion. Results are the pool of 2 individual experiments performed on 2 different EC isolates. **c**-**d** YAP expression in ECs lacking ARHGAP18 (siGAP18) is significantly reduced compared to siControl (siCtl) treated ECs under static (**c**) and 24 h high shear flow (**d**). Western blots of representative experiment, quantification of mean of 3 experiments. (**e**) Total and phospho-YAP protein expression in HUVECs infected with either control (EV) or ARHGAP18 (GAP18) containing adenovirus, measured by Western blot, 6 days after infection. β-actin was used as a loading control. Quantification of densitometric ratio for YAP and phopho-YAP presented as the normalised expression of mean of 3 independent HUVEC lines ± SD. P by paired t-test

In embryonic development ARHGAP18 has been shown to be a downstream effector of YAP [21]. Therefore, we investigated the possible link in mature ECs. Unexpectedly depletion of YAP had no effect on ARHGAP18 protein expression (Fig. 1a). At present we do not have a measurement of ARHGAP18 activity. Therefore, we measured RhoGTPase activity in the presence or following depletion of YAP. Interestingly, depletion of YAP in the ECs resulted in an increase in RhoC activity but no change in RhoA activity (Fig. 1b). In ECs we have previously demonstrated that ARHGAP18 has RhoC but not RhoA activity [8]. We then measured the effect of ARHGAP18 depletion on YAP itself. This resulted in a decrease in YAP expression when the cells were either under static conditions (Fig. 1c) or when subjected to high shear stress laminar flow conditions (Fig. 1d). Further, overexpression of ARHGAP18, although not affecting overall levels of YAP, resulted in an increase in the phosphorylation of YAP (Fig. 1d) [24] suggesting a change in the activity of YAP.

### Loss of alignment with YAP depletion

**Fig. 2:**
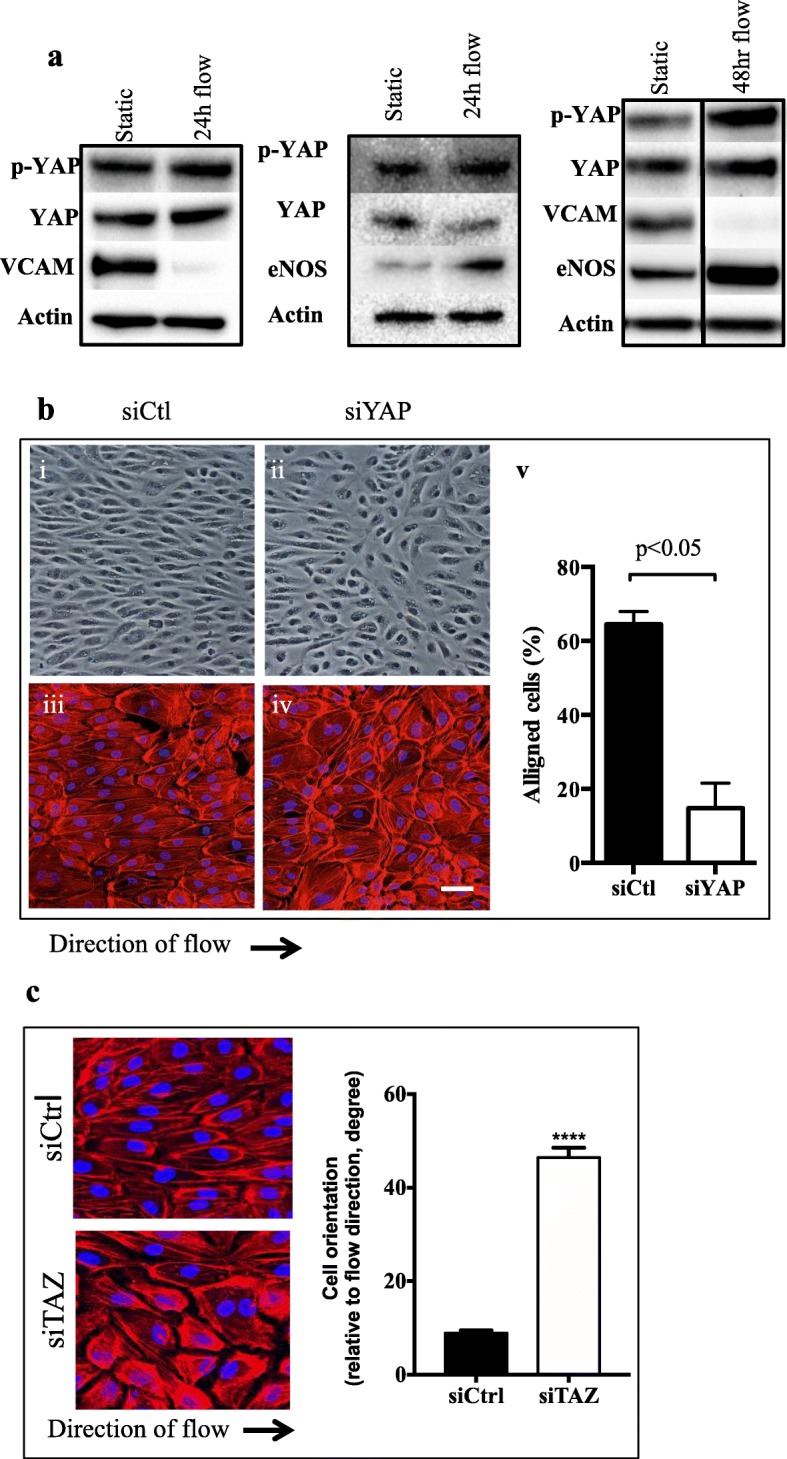
Flow regulates YAP. **a** Flow-induced up regulation of YAP and pYAP at 24 and 48 h high shear flow. Actin given as loading control. Representative of 2 experiments for each time point. **b** Morphological characteristics of cells treated with siRNA. siCtl; (**i** and **iii**) or siYAP–treated ECs (**ii** and **iv**) under high shear flow imaged under bright field or for F-actin staining (red) Scale bar = 50 μm. Direction of flow as indicated. Representative of 3 independent experiments. Quantification of cellular alignment determined by counting the number of cells with actin stress fibers that were parallel to the direction of the major axis of cells. Approximately 1000 cells were counted, from 3 to 4 biological replicates. *p* < 0.05 by paired t-test. **c** Morphological characteristics of cells treated with siCtl or siTAZ. Two experiments *p* < 0.0001. Cell orientation angles with respect to the flow direction were used for the measurement of alignment when cells aligned to flow

We have recently shown that ARHGAP18 is essential for EC alignment under high shear stress laminar flow [8]. Since YAP activity is up-regulated under oscillatory shear stress (OS) [17] and given that ARHGAP18 regulates YAP levels of expression we investigated whether YAP also influences EC alignment. Normal control EC placed under 24–48 h laminar flow show a non-activated phenotype as judged by lack of adhesion molecules such as VCAM1, high expression of eNOS and consistent with that in the literature, high levels of pYAP (Fig. 2a) [16]. Indeed, these cells aligned over the 24–48 h when placed under high laminar shear stress (Fig 2bi, iii). Depletion of YAP by siRNA induced a depletion of > 95% of the protein at 48 and 72 h after transfection (Fig. 1a). There was no change in the viability of the cells. When plated under laminar high shear flow conditions, these YAP-depleted ECs failed to align in the direction of flow (Fig. 2b, ii, iv). Quantification of alignment, based on the number of cells where the actin stress fibres were aligned in the direction of flow, showed a significant reduction in the alignment in the YAP-depleted ECs (Fig 2bv). Depletion of TAZ also results in a failure of the cells to align under flow (Fig. 2c). In this case we quantified the cell orientation, relative to the flow direction, another method for measuring cell alignment. Thus, alignment of EC in the direction of flow is dependent on both ARHGAP18 and YAP/TAZ expression.

### Depletion of ARHGAP18 activates YAP

**Fig. 3:**
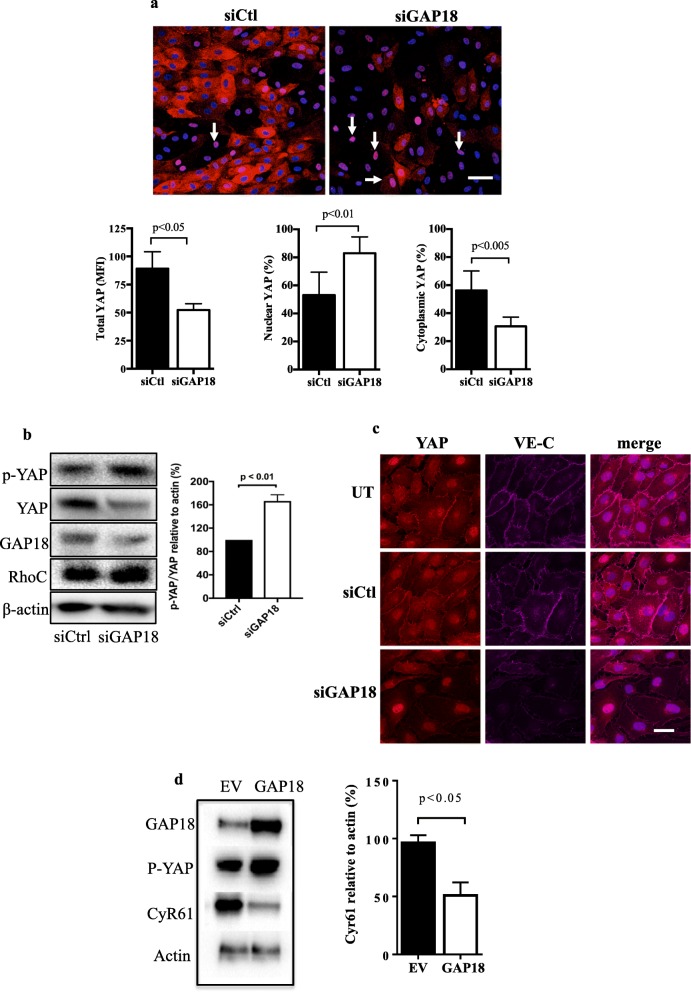
ARHGAP18 depletion results in YAP activation. **a** Cells were treated with siCtl or siARHGAP18 (siGAP18) and placed under 48 h of laminar flow. Representative images showing nuclear YAP (pink, white arrows) in ECs lacking ARHGAP18 under high shear flow. Nuclei (blue). Scale bar = 50 μm. Quantification of MFI for *n* = 3 experiments of total, nuclear and cytoplasmic YAP. **b** Cells were depleted of ARHGAP18 by siRNA and then total YAP or p-YAP measured 72 h later. A representative western blot and quantification of *n* = 3 experiments is shown. **c** ECs were untreated (UT), treated with siCtl or siARHGAP18 (siGAP18) and 48 later imaged for YAP and VE-cadherin expression. **c** Cyr61 protein expression in ECs lysates 6 days after EV or ARHGAP18 (GAP18) infection. β-actin was used as a loading control. Quantification of densitometric ratio for Cyr61relative to actin. Data are presented as the normalised expression of mean of 3 independent EC lines ± SD. p by paired t-test

The lack of alignment under high shear stress laminar flow in both ARHGAP18 and YAP-depleted ECs is similar to that described for EC in disturbed flow [25]. Further in these regions YAP is known to be activated as judged by its nuclear localisation and decrease in phosphorylation [13]. Therefore, we investigated the activation status of YAP following ARHGAP18 depletion, using both localisation and phosphorylation studies. Although ARHGAP18 depletion resulted in a loss of total YAP (Fig. 3c, d), we noted a significant change in its localisation, with an increase in the nuclear but decrease in the cytoplasmic compartments (Fig. 3a). Interestingly, although depletion of ARHGAP18 resulted in a loss of total YAP, there was an increase in expression of phospho-YAP-S127 (Fig. 3b). This was unexpected since such phosphorylation it is often a measure of inhibition of activation. However, phosphorylation of YAP at S127 is also associated with directing the protein to degradation compartments [18, 19]. Previous reports have shown that in stable junctions YAP is associated in VE-cadherin-mediated EC contacts [26, 27]. When ARHGAP18 is depleted we have previously shown a loss of junctional stability associated with a loss of VE-cadherin at the junctions [7], as seen in Fig. 3c. This was associated with an overall loss of YAP from the cytoplasm and particularly a loss of YAP from the junctions. However, with ARHGAP18 depletion, YAP is still apparent in the nucleus (Fig. 3c) consistent with YAP activation and disrupted junctions. In contrast, overexpression of ARHGAP18 resulted in a decrease in CyR61, a downstream pro-inflammatory target gene for YAP (Fig. 3d), suggesting a decrease in YAP activity. Together, these results show that ARHGAP levels impact on YAP activity. We find a depletion of ARHGAP18 results in YAP activation, whereas an increase in ARHGAP18 expression inhibits YAP activation.

**Fig. 4:**
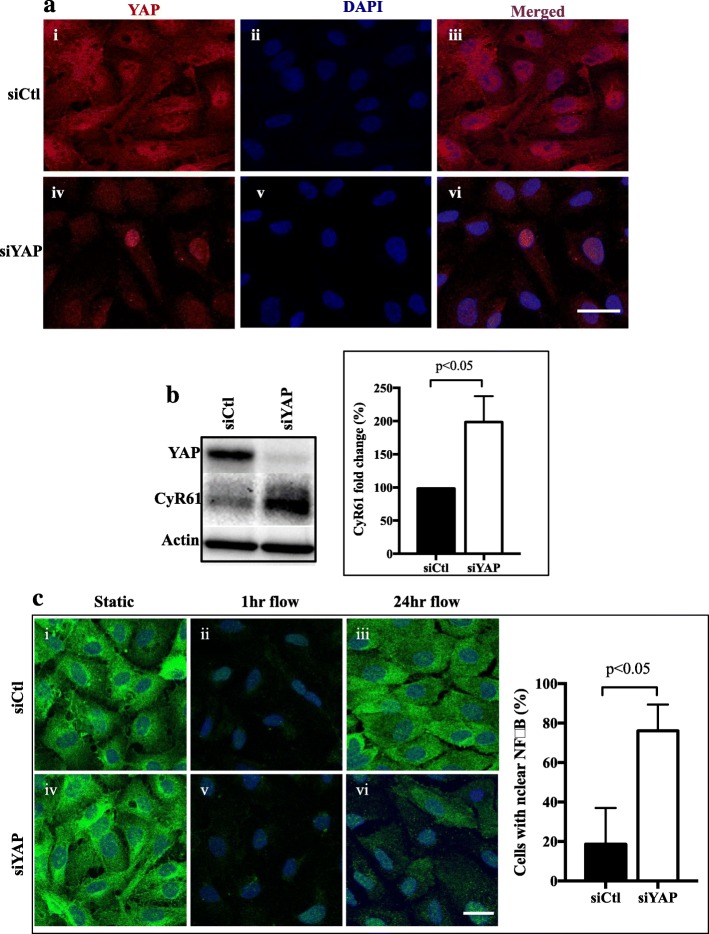
YAP depletion results in its activation. **a** Treatment of ECs with siYAP (**iv**) resulted in 90% knockdown of YAP (red) in the cytoplasm [15] compared to siCtl treated ECs (**i**). Nuclei (DAPI blue). **vi** Detectable amounts of YAP reside within the nucleus (Pink) in YAP knockdown ECs compared to siCtl treatment (**iii**). Scale bar = 25 μm. **b** YAP depletion by siRNA results in an increase in its target gene CyR61 as measured by Western blot analysis. Quantification of *n* = 3 experiments. **c** YAP depletion by siRNA results in maintenance of p65 in the nucleus and a failure to re-localise back into the cytoplasm after 24 under laminar flow, representative images of one experiment is shown and quantification of the amount of nuclear p65 (NFkB) from *n* = 3 experiments. p by paired t-test

Depletion of YAP itself also resulted in its activation. YAP depletion with siRNA, resulted in the loss of total YAP particularly evident from the cytoplasmic portion. However, nuclear YAP was evident at least in some cells, suggesting activation (Fig. 4a). This was confirmed by the increased expression of the YAP downstream target, CyR61 (Fig. 4b). A further indication that the cells are activated with loss of YAP was seen with the nuclear localisation of p65 as a marker of NFkB particularly under laminar flow conditions (Fig. 4c). In control cells, p65 was rapidly lost from the cytoplasm, as a result of transient flow induced changes as previously described for NFkB [8, 28]. However, with 24 h of flow the cytoplasmic localisation was restored consistent with the anti-inflammatory, non-activated phenotype of ECs under laminar flow [9, 25]. In YAP-depleted cells cytoplasmic p65 was also lost at 1 h after flow induction. However, in contrast to that seen in control cells there was a significant failure of p65 to be relocalised to the cytoplasm after 24 h and a significant proportion of cells continued to show nuclear localisation (Fig. 4c), confirming cellular activation.

**Fig. 5:**
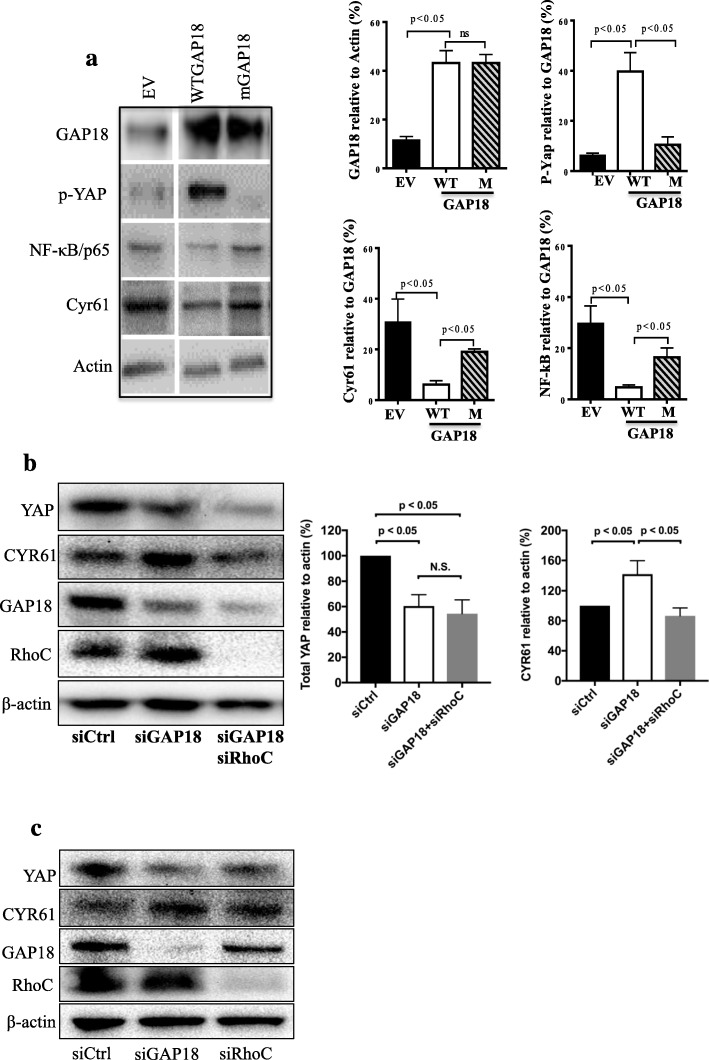
RhoC depletion rescues the ARHGAP18 mediated effects on YAP activity. **a** Overexpression of wild type ARHGAP18 (WTGAP18) or mutant ARHGAP18 (M-GAP18) in ECs and quantification of expression of pYAP, CyR61 and p65. Representative images of one experiment is shown and quantification of the proteins from *n* = 3 experiments. p by paired t-test. Levels of ARHGAP18 and pYAP were calculated relative to actin. Levels of CyR61 and p65 were calculated relative to levels of ARHGAP18. **b** Cells were treated with siRNA to ARHGAP18, then 24 h later treated with siRNA to RhoC and 48 h later, lysates were made and probed for indicated proteins. Representative images of one experiment is shown and quantification of the proteins from *n* = 3–6 experiments is given. **c** Cells were treated with siRNA to ARHGAP18 or RhoC and probed for the indicated proteins. Western from a representative experiment to indicate that RhoC knockdown alone was effective on RhoC and had no significant effect on ARHGAP18, YAP, CyR61 expression

ARHGAP18 shows both GAP dependent and GAP independent functions [11, 29]. The GAP activity of ARHGAP is abolished by a single amino acid substitution in the GAP domain [11]. Overexpression of this mutant ARHGAP18 although producing similar levels of ARHGAP18 expression, failed to increase pYAP expression (Fig. 5a). Although wild type ARHGAP18 suppressed the CyR61 expression, the mutant ARHGAP18 did not show the same level of suppression. Further, while the wild type ARHGAP18 suppressed p65 consistent with the previously described anti-inflammatory function of ARHGAP18 [11], the mutant ARHGAP18 failed to significantly suppress p65. Together, the results suggest that the GAP activity of ARHGAP18 is essential to mediate the regulation of YAP activity.

We have previously shown that ARHGAP18 has activity against RhoC in ECs and that depletion of RhoC rescues the effect of ARHGAP18 knockdown on junctional disruption [8]. Since the results in Fig. 5a Ashow that the GAP activity is necessary for the regulation of YAP and its activation as measured by CyR61 expression, and the results in Fig. 1a show YAP has activity on RhoC and not RhoA, we investigated whether RhoC can rescue the YAP activation. Cells were depleted of ARHGAP18 alone or in conjunction with RhoC depletion. The results show that whereas ARHGAP18 depletion alone resulted in CyR61 increase in expression, depletion of RhoC rescued this effect and reversed the increased expression (Fig. 5b). Loss of RhoC had no significant effect on YAP or ARHGAP18 expression (Fig. 5c).

### YAP is activated in the aorta of ARHGAP18 knockout mice

**Fig. 6:**
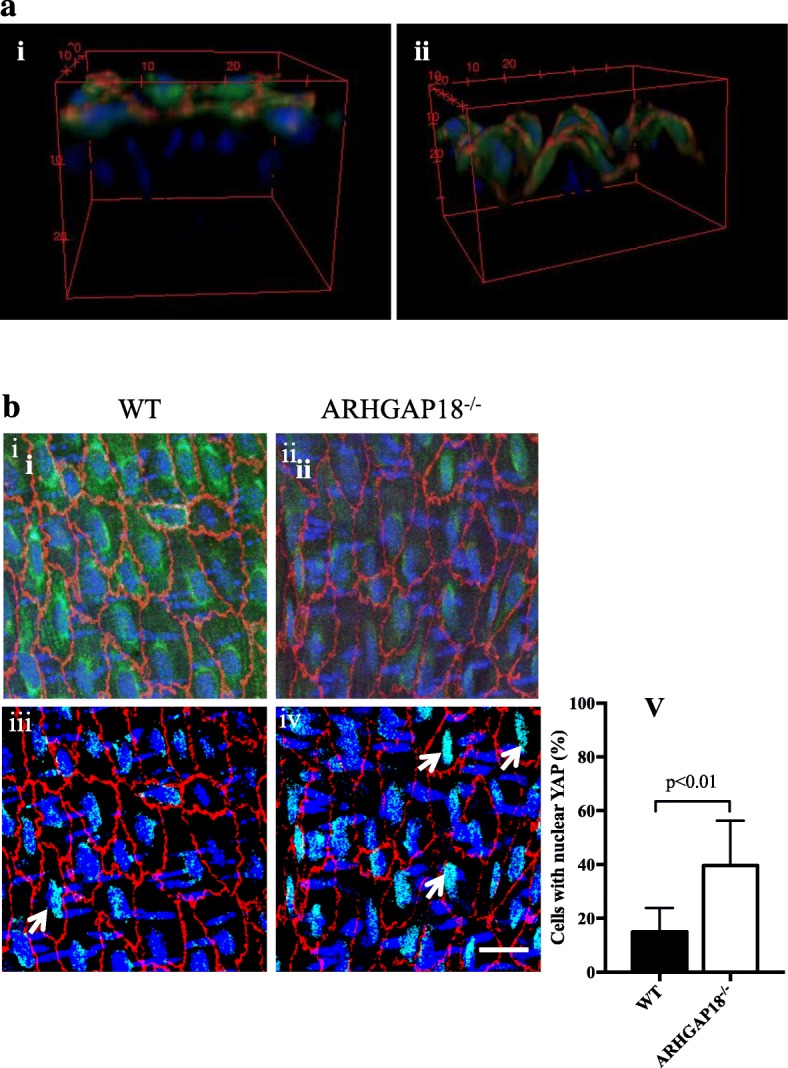
YAP is localised in the nucleus in areas of disturbed flow in ARHGAP18^−/−^ mice. **a** 3D reconstructed images of the endothelium side view of aortic arch (**i**) and thoracic aorta (**ii**), showing YAP expression (green) exclusively on the VE-Cadherin positive EC layer (red), nuclei (DAPI, blue). **b** YAP expression levels in ECs in the thoracic aorta regions in WT (**i**, **iii**) and ARHGAP18^−/−^ mice (**ii**, **iv**). The processed binary images (**iii**, **iv**) taken from WT and ARHGAP18^−/−^ after cytoplasmic YAP exclusion (as given in the M&M) shows increased nuclear localisation in the ARHGAP18^−/−^ (white arrows). Images are representative of 6 independent experiments performed. Scale bar = 20 μm. **v** Percentage of cells with nuclear localised YAP, mean +/− SD, *n* = 6 mice

Finally, we investigated YAP expression in the endothelium of mice, either WT or ARHGAP18^−/−^ mice. The EC layer was identified by the expression of VE-cadherin, and imaging for YAP was performed on this same plane (Fig. 6a). Analysis of the YAP levels in the ARHGAP18^−/−^ mice showed a reduction in the total amount of YAP (Fig 6bi-iv), consistent with our in vitro results (Fig 6bi-iv). To quantitate nuclear YAP, the images were processed by an image calculator, so that all cytoplasmic YAP was excluded and only nuclear YAP was imaged in the endothelium. This showed a significant increase in nuclear YAP expression in ECs from ARHGAP18^−/−^ compared to WT mice (Fig 6bv).

## Discussion

Cell alignment in the direction of flow is a major protective response of ECs to shear stress. The alignment and remodelling are mediated through the dynamic regulation of the family of RhoGTPases [30]. We have previously demonstrated that ARHGAP18 (a.k.a *SENEX)* [10], a negative regulator of RhoC [7], is flow responsive and is essential for the alignment of ECs in the direction of flow [8]. We show here that the ARHGAP18/YAP axis is essential for EC to align in the direction of flow. Loss of ARHGAP18 or loss or activation of YAP impairs this alignment.

The transcriptional co-activator YAP has been linked to control of organ shape, size and alignment by regulation of tissue tension [31, 32] . YAP activity is regulated both by the canonical HIPPO/LATS pathway and also by non-canonical LATS independent Rho/cytoskeleton pathway [15, 32, 33]. In ECs, YAP/TAZ delivers the mechanotransduction cues from the extracellular matrix to control cell survival mediated through Rho and the cytoskeleton [13]. The athero-protective unidirectional shear flow results in a decrease in the activity of YAP in EC mediated through the integrin/RhoA axis [34]. Most recently, oscillatory shear stress has been shown to lead to YAP activation mediated through the integrin alpha 5beta1 [35]. In embryogenesis, ARHGAP18 is an effector of YAP [21]. Here, we show that in mature EC, ARHGAP18 regulates YAP, as demonstrated in both overexpression and knockdown studies of ARHGAP18 and also as seen in the ARHGAP18^−/−^ mice and ARHGAP18 and YAP regulate the mechanotransduction activity in EC. Most interestingly we show that loss of YAP, through either ARHGAP18 depletion or through siRNA depletion of YAP itself, resulted in activation of YAP, defined by a decrease in cytoplasmic YAP levels, nuclear localisation of YAP and through an increase in the YAP target gene, CyR61. This was unexpected as it is in contrast to that previously seen where knockdown of YAP phenocopied the protective effect of laminar flow at least on the cell cycle inhibitor p21 and on the pro-inflammatory cytokine, CCL2 [16, 17]. The shuttling of YAP between the cytoplasm and nucleus is rapid and continuous and its nuclear retention is dependent on a tense cytoskeleton [13]. One possibility is that depletion of ARHGAP18 may alter the YAP shuttling since loss of ARHGAP18 disrupts MTs and actin in EC [11]. Indeed, at least in mesenchymal stem cells, Rho and the actin cytoskeleton are required to maintain nuclear YAP/TAZ [13]. Further, our results are similar to that reported by Yu and colleagues where suppression of Rho retained pYAP [36], in our case suppression of RhoC through ARHGAP18 overexpression results in increased pYAP. In addition, ARHGAP35 encoding the p190RhoGAP has a similar effect on YAP localisation in epithelial cells to that we find in EC and presented here. A loss of a single p190 paralog results in nuclear localisation of YAP and transactivation of genes involved in loss of contact inhibition [37]. Thus, effects on YAP activation are likely to be both cell type and stimulant specific. Most significantly, the activation of YAP was also seen in the ARHGAP18^−/−^ mice where there was a decrease in overall expression of YAP but a nuclear enrichment of YAP in the ECs in the descending region of the aorta. The activation of YAP in these regions in the ARHGAP18^−/−^ mice, is consistent with the lack of alignment under flow and the priming of this region towards a more pro-inflammatory phenotype which we have recently reported [8]. Finally, our results may also be explained by the loss of ARHGAP18 resulting in disruption of VE-Cadherin localisation [7] a state that is known to result in activation of YAP [26]. Indeed, our results show that ARHGAP18 depletion leads to a disruption of the junctions as seen by loss of VE-Cadherin localisation to these regions and a concomitant localisation of YAP to the nucleus.

ARHGAP18 has both GAP dependent and GAP independent activity [11, 29]. Our data, with both the GAP mutant ARHGAP18, as well as rescue experiments (Fig. 5), demonstrate that the GAP activity is essential for the regulation of YAP and that RhoC can rescue the effect of depletion of ARHGAP18. Interestingly, depletion of YAP itself results in increased RhoC but not RhoA activity (Fig. 1b) suggesting that in ECs, RhoC may lie both upstream and downstream of YAP. This is consistent with previous reports showing that RhoGTPases lie both upstream and downstream of YAP. Alternately, it may suggest a feedback loop operating between ARHGAP18/YAP. Further work is required to investigate these possibilities and to delineate the mechanism of activation of ARHGAP18. However, our study highlights that in EC, ARHGAP18 is a major regulator of the RhoC-GTPase system that impacts on YAP and on flow responsive cell alignment.

## Conclusion

We have shown that ARHGAP18 and YAP promote the shape change and athero-protective alignment of ECs in the direction of laminar shear stress. In conjunction with our previous studies we suggest ARHGAP18 is an essential inhibitor of the development of inflammation in the endothelium.

## Data Availability

Not applicable.

## Abbreviations

ARHGAP18: Rho GTPase Activating Protein 18
ECs: Endothelial cells
HUVECs: Human umbilical vein endothelial cells
NFkB: Nuclear factor kappa-light-chain-enhancer of activated B cells
YAP: Yes-associated protein
